# Natural Biocidal Compounds of Plant Origin as Biodegradable Materials Modifiers

**DOI:** 10.1007/s10924-021-02315-y

**Published:** 2021-10-23

**Authors:** Alona Pawłowska, Magdalena Stepczyńska

**Affiliations:** Department of Materials Engineering, Kazimierz Wielki University, J.K. Chodkiewicza 30 street, 85-064 Bydgoszcz, Poland

**Keywords:** Biodegradable polymers, Natural origin modifiers, Biocidal additives, Polyphenols, Phytoncides

## Abstract

The article presents a literature review of the plant origin natural compounds with biocidal properties. These compounds could be used as modifiers of biodegradable materials. Modification of polymer material is one of the basic steps in its manufacturing process. Biodegradable materials play a key role in the current development of materials engineering. Natural modifiers are non-toxic, environmentally friendly, and renewable. The substances contained in natural modifiers exhibit biocidal properties against bacteria and/or fungi. The article discusses polyphenols, selected phenols, naphthoquinones, triterpenoids, and phytoncides that are natural antibiotics. Due to the increasing demand for biodegradable materials and the protection of the natural environment against the negative effects of toxic substances, it is crucial to replace synthetic modifiers with plant ones. This work mentions industries where materials containing natural modifying additives could find potential applications. Moreover, the probable examples of the final products are presented. Additionally, the article points out the current world’s pandemic state and the use of materials with biocidal properties considering the epidemiological conditions.

## Introduction

Modification of polymer materials is carried out to give the desired characteristics to the final products. These features depend on the application. For example, the industry in which the polymer materials will be used or the function they will perform. Modification is the most common way to give unique features and improve selected parameters of finished goods made from polymer materials. The modification changes their properties and internal structure. One of the methods of changing the internal structure of materials is the insertion of modifying additives. In general, it is carried out during the production process. The aspiration to reduce the number of additives gave rise to the search for modifiers that would give more than one new feature to the biodegradable material. Due to the current epidemiological situation, the research of compounds with biocidal properties added to polymer materials is in more dynamic progress than ever before. The biocidal properties of the material are understood as the capability to reduce the number of pathogenic microorganisms under defined conditions [1–4]. The increase of public awareness of environmental pollution, the constant growth of the number of post-consumer waste, and care for human health are observed nowadays. Thus additives should meet the modern criteria and conditions. First of all, they should be non-toxic to human health and the environment [5]. Polymer materials used in the medical, pharmaceutical, and packaging industries are in close contact with the human body or food [6, 7]. The application of polymer materials in mentioned industries may depend on the substances they contain. The additives contained in the material cannot interact with other materials. All of the mentioned conditions concern both modifying additives and polymer matrices.

Biopolymers are the polymers that naturally occur in flora and fauna [8]. They cause no environmental pollution and are completely harmless for the inhabitants of the earth. Moreover, this kind of polymers is obtained from renewable resources which do not destroy our planet. The natural origin of this type of materials makes them biodegradable—susceptible to chemical processes that lead to the decomposition of biochemical substances. The decomposition is done by microorganisms [9]. The biopolymers are well suited for biocomposites (composites that are produced from renewable raw materials) manufacturing due to their biodegradability, no-toxicity, and natural origin.

Biocomposites are promising materials that may be implemented into everyday use. The processing of biocomposites with biopolymer matrix doped with natural-origin modifying additives is a promising field of biodegradable biocomposites. Biocomposites like this are the present development trend in polymer materials. The current literature state helps to create a classification of modifying additives of natural origin which contain organic substances.

This article presents already used modifiers and those having the potential to be used in the production of polymer materials that exhibit biocidal properties. The synthesis, properties, and application of natural modifiers of plant origin are discussed. The basic groups of polyphenols and selected compounds of natural origin such as some phenols, naphthoquinones, triterpenoids, or phytoncides are presented.

## Biopolymer Matrices

Polymer might be called biopolymer if it is biobased (produced by the living organism) or/and biodegradable. They are divided into two groups. The first group of biopolymers (natural) is obtained from living organisms, while the second one (synthetic) is produced during the polymerization of selected compounds contained in renewable resources [10–12]. Moreover, the natural biopolymers group consists of two subgroups: polysaccharides and proteins, while the synthetic biopolymers group is divided into degradable and non-degradable biopolymers [10]. Most of the representatives of mentioned groups are used as materials for biocomposite matrices. Due to the current ecological threat resulted from residual plastic waste the creation of completely biodegradable composites is crucial. It is possible, however, every part of the composite should be biodegradable. Therefore, the application of natural plant modifiers in biocomposites is an excellent solution for environment-friendly materials.

### Chitosan

Chitosan belongs to polysaccharides. This biopolymer is sourced from chitin—one of the most common polysaccharides in the world. It occurs in marine shellfish, insects, mushrooms, and yeast. The highest percentage content of chitin has been observed in shells and tails of crabs, shrimps, and lobsters. Hence, chitosan could be “recycled” from seafood waste. This method of chitosan extraction could improve current environmental conditions [13–15].

Chitosan is known as a semicrystalline polymer material with various types of crystal structures (polymorphism). It is a biodegradable, biocompatible, and renewable polymer material with antioxidant properties. Moreover, chitosan is non-toxic for humans and bioactive against selected microorganisms and viruses [15, 16]. Material is soluble in acid solution and non-soluble in the majority of solvents. Its hydrophilic properties promote the ability to create films. However, the hydrophilicity of this material has its drawback: it leads to material swelling in water. Hence, material modification is advised. Furthermore, it is known that chitosan is susceptible to modification [17].

This biopolymer is used for environmental protection purposes (e.g. water purification) [17]. In the agricultural industry, chitosan is applied as a biostimulator that promotes plants growth and their defense mechanisms. As a seed coating material chitosan improves its germination rate [18]. Due to its biological activity, it is used in the food and food packing industries as a biopreservative that extends the shelf life of products [19, 20]. Material has potential application in the medical industry due to its unique (e.g. wound healing) properties. Chitosan could be applied as surgical sutures or in bones and dental prosthetics. According to the hydrating properties of the compound, it is could be used as a material for contact lenses [15, 21].

### Starch

Starch is another natural polymer that belongs to polysaccharides [22]. It occurs in plant roots, tubers, and fruits. The main sources of starch are cereals and potatoes. Although it could be extracted from certain varieties of pea and lily [23, 24]. Starch consists of homopolymers—amylase and amylopectin. Amylase is soluble, while amylopectin is non-soluble [8, 22]. Wet grinding, drying, and sieving are the main ways to obtain starch [24].

Energy storage is the main function performed by starch in various plants [25]. Starch is a hydrophilic biodegradable polymer that is non-soluble in cold water and soluble in diluted solutions of acids and bases. It is known that the mechanical treatment (milling) of starch improves its solubility [22, 26, 27]. This polymer is renewable, biocompatible, and biodegradable [24]. Its presence in polymer materials increases their biodegradability [25]. According to Syafiq et al. [28], the mechanical properties of the starch-based films are comparable to currently used plastics. Besides this, the starch films have an advantage—they are edible. Moreover, starch is a low-price polymer [24].

According to the non-toxicity of starch and its unique polymer properties, it is applied in many industries [24]. However this polymer is mainly used in textile [29], pharmaceutical [24], paper [30], printing [31], and cosmetic [32] industries. This polymer would be an excellent material for the production of biodegradable disposables if it exhibited hydrophobic properties [25]. The wide range of starch modification methods enables its application in the food industry [33].

### Zein

Zein which belongs to proteins is one of the most frequently used biopolymers from renewable sources [34]. It is obtained only from maize: corn gluten meal (CGM), distillers dried grains (DDG), and dried milled corn [35, 36]. It is the main protein that occurs in corn endosperm cells and its percentage content varies from 35 to 65% [35, 37].

It is a biodegradable and biocompatible material that exhibits hydrophobic, antioxidant, and mucoadhesive properties [38–41]. The solubility of zein depends on the solvent: it is insoluble in water, while anionic detergents, alcohols, and urea (only in high concentration) dissolve it [35]. Solvent parameters (such as pH and temperature) affect zein structure [42]. According to Arvanitoyannis et al. [43] zein is a brittle material and this characteristic has a negative effect on its structural properties.

Zein is used in the textile, food, and biomedical industries [35]. According to Gonçalves et al. [44], zein-coatings improve the hydrophobic properties of cotton. Due to the United States Food and Drug Administration (FDA) zein is generally recognized as safe (GRAS) [38]. Hence, it is widely used in industries where this polymer could have close contact with the human body. Zein-based edible coatings which contain various antioxidant modifiers are used as food biopreservatives. Zein is applied as a component of adhesives [35]. It is known that the bioactive agents could be encapsulated with this biopolymer [38, 45]. Zein is applied as a material for gene, drug, and vaccine delivery. Tissue engineering is another industry where zein could be potentially used [41]. It could be also implemented as a film and coating material for materials engineering uses. According to the unique properties of zein, it has the potential to replace currently used polymers with the petrochemical origin [42].

### Gelatin

Gelatin is another protein biopolymer [46, 47]. It is obtained from animal skin, bones, cartilages, connective tissues, and fish scales. All the mentioned sources contain collagen [8, 46, 48]. Nowadays, the main source of gelatin is cattle and a pig skin. However, these kinds of animals are suffering from various infectious diseases. Therefore, the alternative sources of gelatin are in constant search. For example, almost one-third of fish waste is skin, scales, and bones which could be used for protein extraction and further gelatin production [25]. It is one of the sustainable ways of waste management which could improve the current ecological state.

According to the natural origin of this polymer, it is completely biodegradable and biocompatible. Gelatin exhibits bioactive (especially antimicrobic), antioxidant, and crosslinking properties [46]. The organic solvents dissolve this material [49]. Gelatin prevents recrystallization and promotes adhesion. However, its adhesive properties depending on the viscosity of the solution. Gelatin is a promising biopolymer for materials engineering applications because of its ability to form films and foams [50]. The fish gelatin is a strong rival to mammal one due to their similar properties. However, the characteristics of fish gelatin depend on the species of fish and the extraction conditions [51].

This biopolymer is widely used in the pharmaceutical, medical, cosmetic, and food industries due to its biocompatible properties [8, 25]. Gelatin is a feedstock for capsules production [49]. Medical applications of material cover mainly tissue engineering (especially tissue regeneration) [52]. Gelatin-based face masks are widely used by consumers [53]. It is known that this polymer is applied as a compound of lotions and creams [54]. The tasteless edible films and encapsulating materials made from gelatin are used in the food industry [49, 55]. Gelatin-based biofilms with improved mechanical properties and water resistance are in current research [55]. Moreover, it is applied as a photographic emulsion and surface modifier [46, 55–58].

### Polylactide (PLA)

PLA is classified as a synthetic degradable polymer [10]. PLA is obtained from lactic acid which is mainly isolated from sugar beets, potatoes, and corn [59–61]. There are two main methods of PLA production: polycondensation of lactic acid and ring-opening polymerization of lactide (extracted from lactic acid) [62, 63]. According to Su et al.’s [64] predictions, the amount of produced PLA will increase in the next 2 years.

PLA is a thermoplastic polymer, its properties (clarity and rigidity) are similar to polystyrene (PS) [65]. However, the melting temperature (T_m_) of PLA is higher and riches 180 °C [61]. Another advantage of PLA is a lower greenhouse gas emission compared to PS [66]. It is completely biodegradable, dissolves in organic solvents, and swells in a wide range of solvents. The water absorption tendency of PLA affects negatively its degree of crystallinity [61, 67]. The toxicity of polymer is low [68].

According to the biocompatibility of PLA it is widely used in healthcare (e.g. medical and drug) and cosmetic industries. Material is used in prosthetics, orthopedics, reconstructive surgery, tissue engineering and so forth. The PLA microcapsules and microspheres are used to reach the effect of prolonged drug release. Cosmetic application of material includes microbeads, face masks, and tissues [68–72]. Due to its relatively high mechanical strength, it is used in the food-packing industry as a vegetable packaging, shrink films, and food trays material. Moreover this it is used for paper bags lamination [73]. The PLA-based packing materials are an environmentally friendly solution that could replace the packaging with petrochemical and non-degradable origin [74, 75]. Another industry of PLA application is the textile industry. PLA fibers are similar to PET fibers, however, the first ones have several advantages: they are more hydrophilic, have better self-extinguishing parameters, and prevent the multiplication of bacteria. Hence, it is used in clothing, towels, wipes, and filters. Due to sustainability and environmental friendliness, PLA is applied in geotextiles [76]. Moreover, PLA is used for environmental purposes as a sorbent that disposes harmful contaminants contained in water. Additionally, PLA takes part in bioremediation—a technique that uses microorganisms to remove environmental impurities [77].

### Polyhydroxybutyrate (PHB)

PHB is another biopolymer that belongs to the degradable biopolymers subgroup [10]. It is produced by various bacteria and microalgae under certain stress conditions (e.g. carbon excess; oxygen, nitrogen, or phosphate deficiency) and performs a storage function [12, 78, 79]. Bacteria and microalgae are the most common forms of life in the world. However, the cultivation and harvesting of these microorganisms is limited due to the expensive equipment used in these processes [12]. According to Sirohi et al. [80], PHB could be isolated from by-products created during production processes in agricultural, dairy, and food industries.

Certain properties (e.g. physicochemical) of PHB are comparable to fossil fuel-based polymer materials. Despite this, the high biodegradability and biocompatibility of this polymer are known. This fact makes PHB a potential alternative to currently used petroleum-based polymers. However, the high production costs of biopolymer are the main obstacle for its implementation into the industry for commercial purposes. It creates no environmental pollution due to the no-toxicity of its degradation products [78–82]. PLA is characterized as a high crystallinity (degree of its crystallinity rages from 50 to 70%) polymer with low water vapour permeability [79, 83–85].

PHB is used in aquacultural, medical, and tissue engineering industries. Moreover, biopolymer is used for equipment production [79, 85]. PHB is applied as a material for the wound dressings and microspheres used in drug delivery systems. Tissue engineering applications of biopolymer and its composites cover sutures, screws, bone plates, staples, rivets, tacks, etc. In the aquacultural industry, it is used as an anti-adhesive agent against shellfish pathogens. While in the agricultural industry it could be applied as an antifouling compound [85]. Also, it is used as an additive for paints and coatings that cause no environmental pollution [86]. It hardly interacts with food, hence it has potential application in the food packing industry [87].

## Classification of Polyphenols

Polyphenols are organic compounds and plant secondary metabolites i.e. final products of enzymatic reactions which occur as a result of metabolism in plants [8, 88]. Polyphenols are present in fruits, seeds, roots, bark, stalk, timber, and leaves of numerous plants. They are divided into phenolic acids, lignans, stilbenes, and flavonoids [89]. This classification is based on the number of phenolic groups contained in the phenolic ring and also on the method of aromatic rings combining. Each group additionally includes over a dozen subgroups. More than 8000 polyphenol compounds have been discovered [90, 91]. The majority of polyphenols are compounds of products that play an essential part in the basic human diet. The well-known properties of polyphenols make them interesting modifying additives of biocomposites [92–94]. The next parts of the article perform a detailed analysis of mentioned groups of polyphenols.

### Flavonoids

**Fig. 1:**
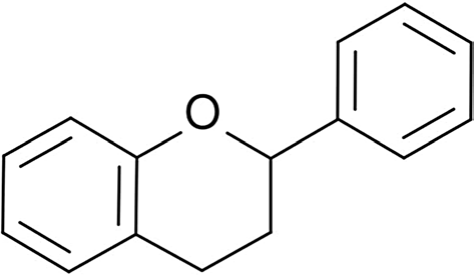
Chemical structure of flavonoids [99]

The best-known group of polyphenols is flavonoids (Fig. 1)—compounds that dye flowers, fruits, and drupes of plants. Flavonoids are the biggest group of polyphenols that includes over 8000 compounds [95, 96]. The number of discovered compounds is constantly increasing [97]. These compounds perform plenty of functions: protecting plants from ultraviolet radiation (UV) damage, creating a biological protective barrier, and exhibit biocidal functions against microorganisms. Furthermore, flavonoids are known as natural antioxidant compounds [98]. Representatives of flavonoids such as flavanones, flavones, flavonols, and isoflavonoids are also well-known for their biocidal properties.

Flavonoids are the promising modifiers of polymer materials due to mentioned properties. These modifiers can be used in the building industry as compounds of products that are exposed to UV radiation (windows, gutters, and other elements manufactured from polymer materials). Increased resistance to UV radiation may improve the aesthetic values which getting worse over time. Window or door elements such as handles doped with biocidal modifiers compounds like flavonoids are necessary in public spaces, especially in current epidemiological conditions. This solution may decrease the number of microorganisms embedded in elements of public usage. In the future, it may reduce the number of infections with various diseases. This group of compounds and their applications are discussed in section ‘Classification of Flavonoids’.

### Phenolic Acids

**Fig. 2:**
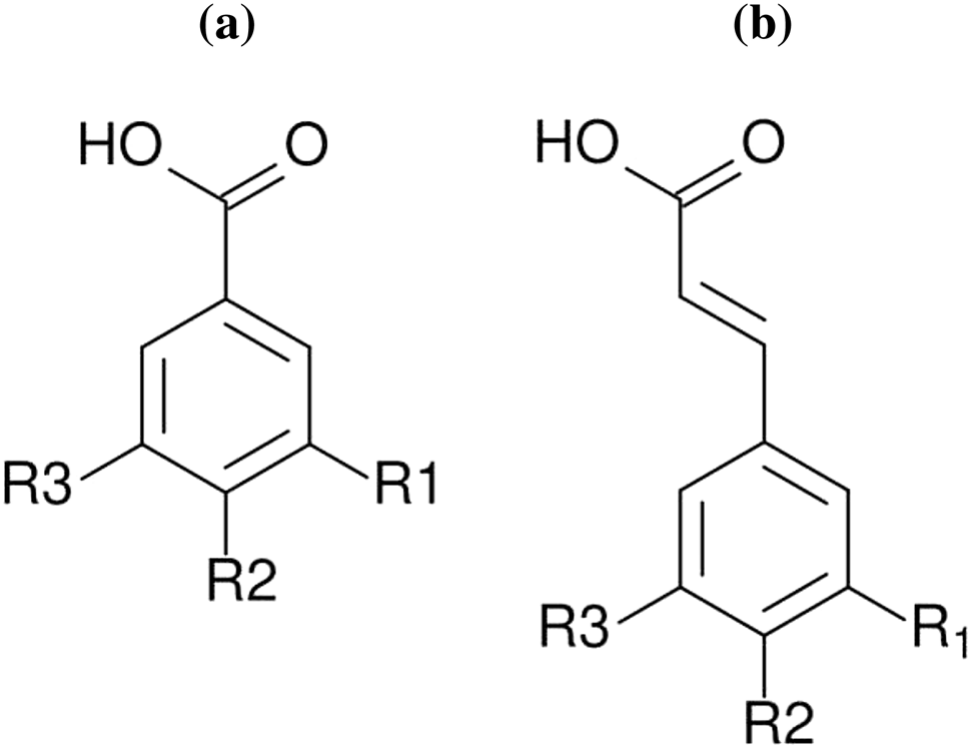
Chemical structures: hydroxybenzoic (**a**) and hydroxycinnamic (**b**) [115]

Phenolic acids are the subgroup of polyphenols that contains carboxyl and hydroxyl groups. Phenolic acids can be divided into two groups: the first group contains hydroxybenzoic structures (Fig. 2a) and the second group—hydroxycinnamic (Fig. 2b). Phenolic acids naturally occur in fruits, vegetables, and grains. They are found as compounds in free form (not connected with other compounds) and as connected form. The last form is connected by ether, ester, and acetal bonds with molecules that perform building functions in plants [100]. Proteins, cellulose, and lignin are responsible for these kinds of functions in plants. Phenolic acids also occur in the form that is connected with polysaccharides (starch). They take part in the synthesis of proteins, nutritional and allelopathic processes. During the allelopathic processes, the toxic substances produced by plants are releasing into the environment. The research confirms that substances performing allelopathic functions are also natural bio-stabilizers—substances that inhibit cell division processes (multiplication) of pathogenic microorganisms [2]. These compounds are contained in cucumber and onion [101–105]. Phenolic acids as modifiers of polymer materials may be potentially applied in conditions with a high risk of microorganisms invasion. Products that are used in humid conditions should perform both exploitative and biocidal functions since humidity promotes the multiplication of microorganisms. Phenolic acids fulfill these conditions and can be used as modifying additives of materials used in water transport systems.

The caffeic, gallic, vanillic acids are the representatives of this group [106]. Caffeic acid connected with chitosan exhibits anti-tumor properties what makes it a potential anti-cancer agent [107]. Zein-based and PLA-based coatings which contain caffeic acid can be used in the food packing industry. PHB/gallic acid nanofibers with antibacterial properties are a novel material for the food packing industry. Chitosan-based mats doped with gallic acid and vanillic acid grafted chitosan (as a wall material) are used for food encapsulation. These materials exhibit antioxidant properties [108–112]. In materials engineering, phenolic acids are used as UV stabilizers in biopolymers (PLA + vanillic acid) [113]. Completely degradable nanoparticles made from PLA which contains caffeic acid could be potentially applied in various industries [114].

### Lignans

**Fig. 3:**
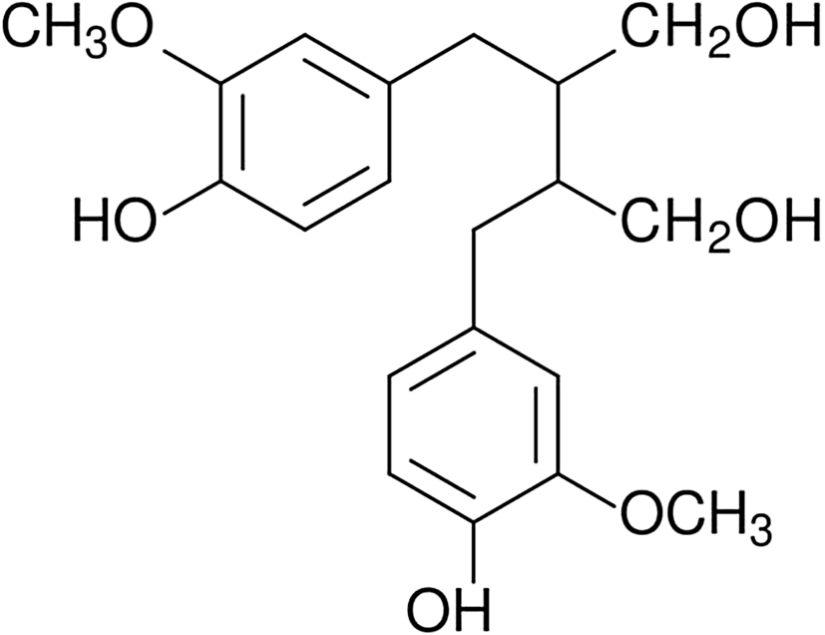
Chemical structure of lignans [120]

Lignans (Fig. 3) are the phenylpropane dimers that are classified as phytoestrogens—the plant origin hormones. Linseed is one of the richest sources of lignans. These phytoestrogens control the growth and development of linseed and also take part in its protection against the harmful effect of UV radiation. They exhibit antifungal and antiparasitic activity. Moreover, lignans are known for their strong antioxidant properties. The solubility of these compounds in the essential oils and resins is high [98, 116]. This property can be used in the field of materials engineering during resin-based materials processing. The biocidal and antioxidant properties of these materials would be increased. The additives that improve more than one property of the biodegradable materials (e.g. mechanical strength and microbiological resistance) are currently searched.

Certain lignans (phyllanthin and silymarin) are implemented into biopolymers. Chitosan-based microcapsules with phyllanthin have a potential application in the pharmaceutical industry [117]. Cellulose biocomposites containing zein/silymarin nanoparticles could be used in food packing. The strong antioxidant properties of this packaging elongate the shelf life of products packed in it [118]. Silymarin improves the resistance of biopolymer blends (PLA/PHB) on thermo-oxidative degradation. Therefore it could be applied as a thermooxidative stabilizer in materials engineering [119].

### Stilbenes

**Fig. 4:**
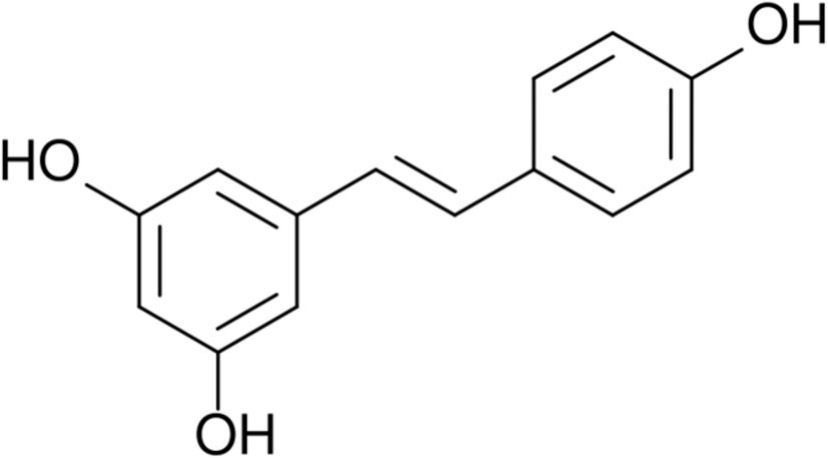
Chemical structure of stilbenes [120]

Stilbenes (Fig. 4) are the organic compounds contained i.a. in berries, grapes, and nuts. These substances have a complicated structure. They exhibit antioxidant and antimicrobic properties and have the ability to polymerization. The antioxidant mechanism of stilbenes is based on the stimulation of proteins and enzymes contained in plant cells. Stilbenes are not widely spread and occur only in 30 plant species. In the years 1995–2008 approximately 400 new stilbenes were discovered [98, 121]. This group of substances exhibits both biocidal and antioxidant properties. The presence of these modifying additives could increase the service life of final products which could be exposed to pathogenic microorganisms and adverse climatic conditions (e.g. UV oxidation).

The resveratrol and piceatannol belong to stilbenes. Biopolymer-based nanoparticles loaded with resveratrol have a potential application in the drug industry. The delivery of resveratrol in nanoparticles improves its solubility in the human body [122, 123]. Biopolymers/resveratrol materials are used as repairing scaffolds in tissue engineering [124]. The gelatin/zein mats and pectin/gelatine films containing resveratrol are used as an active packaging material that elongates the shelf life of food [125, 126].

It is known that resveratrol improves the photo-oxidative and thermal stability of PLA and could be applied in materials engineering [127]. Piceatannol is a stilbene with strong anti-cancer, anti-viral, anti-inflammatory, and antioxidant activity which is used in the pharmaceutical industry. The chitosan-PLA nanoparticles and zein nanospheres have a potential application as drug carriers for the piceatannol [128, 129].

## Classification of Flavonoids

### Flavanones

**Fig. 5:**
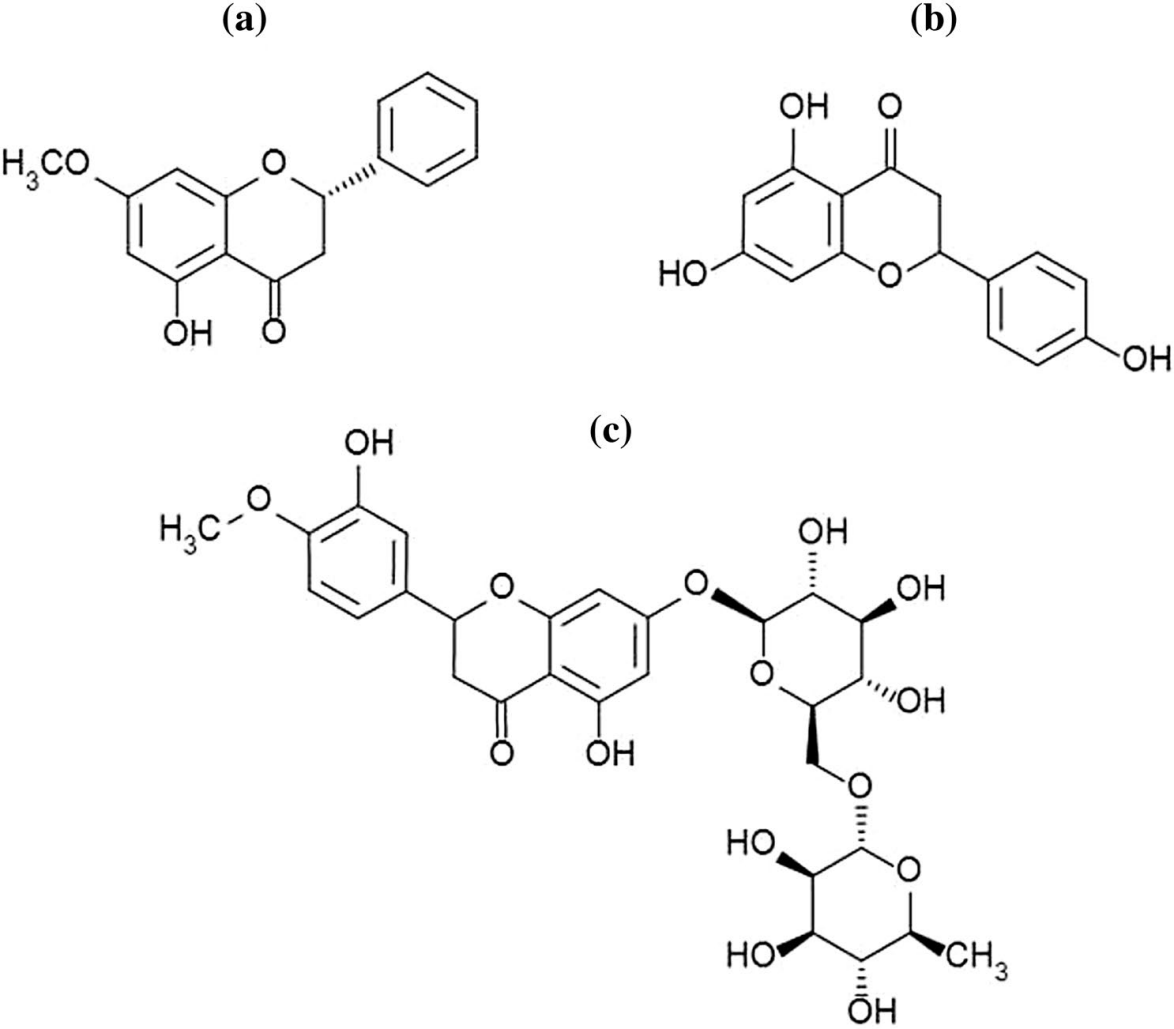
Chemical structure of: pinostrobin (**a**) [131], naringenin (**b**) [149], and hesperidin (**c**) [150]

Flavanones is a small subgroup of flavonoids that takes a big part in medicine. Citrus fruits are the richest source of flavanones. Citrus peel has the greatest concentration of these active substances. Due to their bioactive properties (effecting on human health), citrus fruits extracts are used as well as immunostimulants, preservatives and cleaning agents [130]. Pinostrobin, naringenin, and hesperidin are the most known flavonoids (Fig. 5).

Pinostrobin is obtained from plants *Renealmia alpinia* and *Alpinia zerumbet* [131, 132]. It is the dominant polyphenol contained in certain propolis species [133]. Due to the research described in [134], this substance has a biostatic effect against *Helicobacter pylori* and *Herpes simplex virus* type I.

Chitosan/sodium alginate nanoparticles doped with pinostrobin could be used in the pharmaceutical industry as an anticancer drug [135]. Biopolymer films doped with propolis extract have a potential application as an active packaging material [133].

Naringenin contained in pomegranate juice is a naringin derivative. This compound caused the characteristic bitter taste of pomegranate [136, 137]. Naringenin is also contained in peach drupels, citrus fruits, and tomatoes. Apart from many anticancer properties, naringenin exhibits biostatic activity against *H. pylori* strain and inhibits the enzymes secreted by it [138]. Moreover, this compound exhibits antioxidant and anti-inflammatory properties and also can be used as the agent that inhibits the development of the SARS-CoV-2 virus [139].

Chitosan nanoparticles loaded with naringenin are used in the pharmaceutical industry due to the anti-cancer properties of naringenin [140]. Biopolymer nanoparticles doped with naringenin increase the water solubility of flavanone. This solution has potential application in drug delivery systems [141]. The effectiveness of chitosan-based nanoemulsions doped with naringenin in skin injuries treatment has been proved [142].

Hesperidin like naringenin is contained in citrus fruits and is active against some types of viruses such as *Herpes*, *Poliomyelitis*, and *Paramyxovirus.* Due to the latest research that shows low cytotoxicity of hesperidin, it can be used as an active compound of antivirals against coronaviruses [143–145].

The biopolymer-based hydrogels containing hesperidin in the concentration of 10% could be used as a wound healer agent [146]. Gelatin films with chitosan nanoparticles doped with hesperidin have been considered as an active packaging material [147]. Biopolymer-based materials with hesperidin have a potential application in the food packing industry due to their antioxidant properties and environmental friendliness [148].

### Flavones

**Fig. 6:**
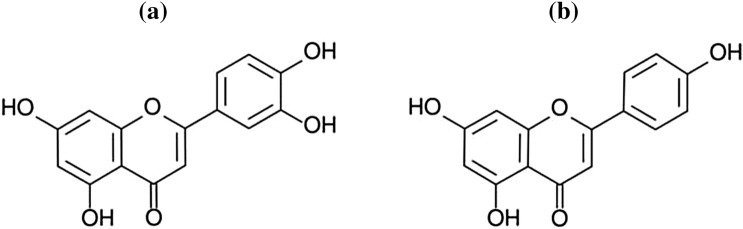
Chemical structure of: luteolin (**a**) [159], apigenin (**b**) [149]

As well as previous group flavones are contained in citrus fruits and their juices [151]. The biological activity was observed in luteolin and apigenin which are classified as flavones (Fig. 6). Luteolin is contained in red onion, kohlrabi, lettuce, arugula, carrots, red and yellow peppers, beetroot, green beans, and spinach. The mechanism of biocidal activity is based on the inhibition process of DNA (nucleic acids are built of nucleotides connected with phosphodiester bond) polymerase [8]. Luteolin exhibits biological activity against the flu virus, *Herpes* virus, and some *Propionibacterium* and *Staphylococcus* bacteria [152, 153]. It is biostatic against *Chlamydia pneumoniaem*, *Trichophyton rubrum*, and *T. mentagraphytes* bacterias. The effectiveness of luteolin against some fungi is comparable to the effectiveness of ketoconazole which is classified as an antifungal drug [152].

Luteolin is used in the pharmaceutical industry, however, the low absorption (bioavailability) in the human body is one of its disadvantages. The biopolymer drug carriers (zein, chitosan, etc.) make it more bioavailable [154–156]. The elongated antioxidant activity of luteolin encapsulated with starch nanoparticles is proved. This kind of nanoparticles could be used in both the drug and food industries [157]. The antioxidant and biocidal properties of luteolin make it an interesting additive for chitosan-based active packing films [158].

Apigenin is contained in i.a. the chicory, pak choi cabbage, and red onion [160]. Clinical trials prove the biostatic activity of apigenin against SARS-CoV-2 and its anti-HIV effect that was similar to the effect of the nelfinavir—the HIV drug [139]. Due to this observation, the substances used in the treatment of HIV can also be applied in cases of coronavirus infection. Apigenin is a natural antioxidant and this property can increase the service life of polymer materials. The cosmetics with an antioxidant effect are in high demand, therefore the apigenin can be potentially implemented in this industry [149]. Luteolin and apigenin constitute a new interesting application—as active ingredients of polymer materials with biocidal properties.

As well as luteolin, apigenin is a flavone with low bioavailability. This property could be improved with its introduction into zein/lecithin nanocomposite. This material has a potential application in the pharmaceutical, cosmetic, and food industries [161]. Chitosan also enhances the solubility of apigenin and could be used as its carrier in drug delivery systems [162]. The apigenin hydrogels based on biopolymers [gelatin, chitosan, and polyethylene glycol (PEG)] promote diabetic wound treatment. Hence, its prospects in diabetic skin injuries therapy is huge [163]. Chitosan-based nanogels loaded with apigenin stop the cancer cells proliferation, therefore the potential application of these materials in oncology is justified [164]. Starch-apigenin complex has a potential application as a supplement supporting stable glucose level in blood. In materials engineering, apigenin could be used as a thermal stabilizer of starch [165].

### Flavonols

**Fig. 7:**
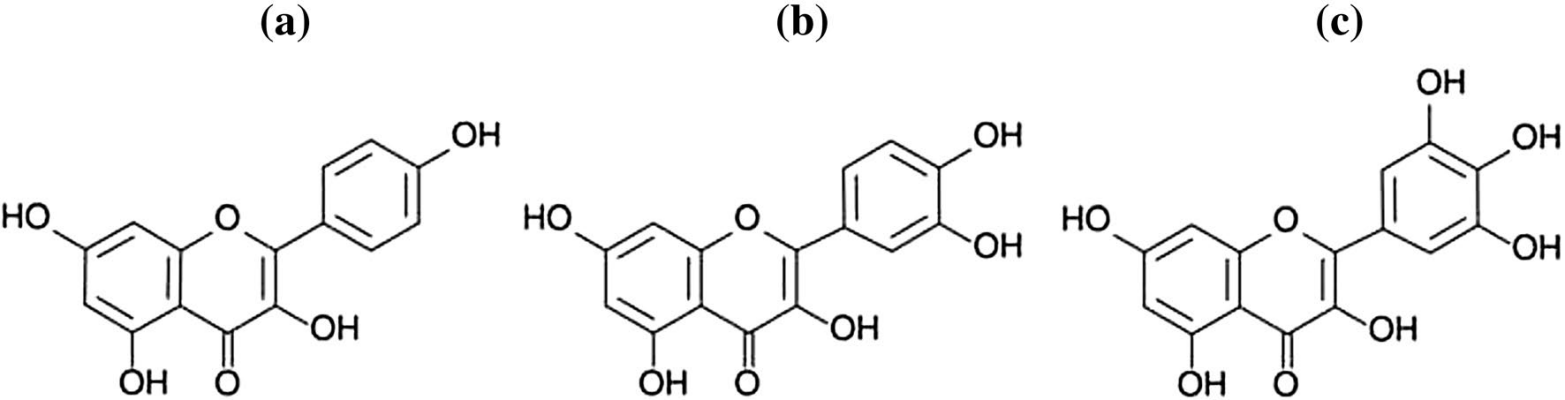
Chemical structure of: kaempferol (**a**), quercetin (**b**) [168], and myricetin (**c**) [169]

Kaempferol, quercetin, and myricetin are the best known and the most common flavonols (Fig. 7) [160, 166]. This group performs different functions such as photoprotection, i.e. protects plants against the harmful effects of UV and parasites, gives plants colour, and also prevents oxidation processes [167]. The biological activity of flavonols depends on their chemical structure and the presence of hydroxyl groups [166].

Kaempferol occurs in many plant species, however the highest content of this substance is found in several plant species: acacia, saffron, aloe, ginkgo, goatweed, leaf flower, and rosemary [170, 171]. Among berries, kaempferol occurs in blackcurrants, gooseberries, and strawberries [160, 172]. This substance also exhibits antioxidant and antimicrobial properties [170]. Kaempferol is a nontoxic substance and has the ability to inhibit inflammatory processes caused by *H. pylori* which take place in the human body [172, 173].

Zein nanoparticles coated with alginate and chitosan are used for kaempferol encapsulation in order to increase its absorption in the blood. This form of kaempferol administration is a prospective solution for drug delivery systems [174]. Zein-kaempferol coatings improve the mechanical and bioactive properties of scaffolds, thus their tissue engineering application is justified [175]. The biopolymer-based membrane doped with kaempferol could be potentially applied as an infected wound dressing [176]. The study of gelatin nanoparticles doped with kaempferol confirms that it could be used as an eye drops for certain eye diseases [177]. The lecithin/chitosan nanoparticles loaded with kaempferol has a potential application as an antifungal agent [178].

Quercetin is a substance with antioxidant properties. It stimulates the human enzyme system which is responsible for metabolic processes [96]. This substance has been applied in silver nanoparticles processing and replaced widely used reducing agents which pollute the environment. Silver nanoparticles with biocidal properties that are obtained with quercetin are called “green” due to the ecological method of their production [179, 180]. Quercetin exhibits biostatic properties and the activity of these properties depends on the type of bacteria. It is a strong agent that inhibits Gram-positive bacteria, however, its activity against Gram-negative bacteria is weak. Research proved that quercetin inhibits the growth of several bacterial strains: *Escherichia coli*, *Staphylococcus aureus*, *Pseudomonas aeruginosa*, and *Salmonella enterica* [181]. The literature overview reveals that both quercetin and kaempferol are bioactive against *SARS-CoV-2* and inhibit metabolic processes of this virus type [139]. This property is crucial in the current pandemic situation. Previously mentioned modifiers have a potential application as biocomposites additives. These modifiers could limit the spread of the pathogenic virus on the surface of materials. Due to their properties, the biocomposites that contain this type of modifying additives could be applied in the public spaces and medical industry.

Zein is used for quercetin encapsulation and as a drug carrier. Due to the bioactive and antioxidant properties of the material, it could be applied in the pharmaceutical, healthcare, food, and food packing industries [182–185]. The potential biomedical applications are based on the implementation of quercetin encapsulated with biopolymers [186, 187]. It has been proved that biopolymer-based hydrogels containing quercetin regenerate bones. Therefore, these hydrogels could be applied as scaffolds in tissue engineering [188]. Chitosan, chitosan/gelatin, and starch/gelatin films loaded with quercetin elongate the shelf life of food due to the above-mentioned properties. Hence, it is well-suited for food packaging applications. Additionally, these films are edible, which makes them even safer for humans [189–193]. The presence of quercetin in PLA-based films makes them interesting materials for active packaging due to the antibacterial activity [194]. Lecithin/chitosan nanoparticles doped with quercetin have a potential application in functional food (food that besides its nutritional value prevents diseases or supports health) production [195]. The thermal stability of starch doped with quercetin was greater than the pure starch one. This fact suggests that quercetin could be potentially used during polymer processing [196].

Another flavonol is myricetin that occurs in parsley, marigold, berries, grapes, oranges, broad beans, herbs, wine, and tea [197–200]. The characteristic feature of this compound is the high melting point—357 °C. The activity of myricetin against *S. aureus* has been reported in [153]. It is a natural antioxidant compound that neutralizes free radicals. The application of this substance in various industries is complicated due to its physicochemical properties, which are poorly understood so far [198].

Chitosan, chitosan-based, and starch materials could be applied as myricetin carriers used in the therapy of various diseases [201–203]. Certain biopolymers improve the bioavailability of myricetin [204]. Chitosan loaded with flavonols (myricetin, kaempferol, and quercetin) is considered to be an excellent active packaging material due to its modified properties (antimicrobial, antioxidant, etc.) of chitosan [205].

### Isoflavonoids

**Fig. 8:**
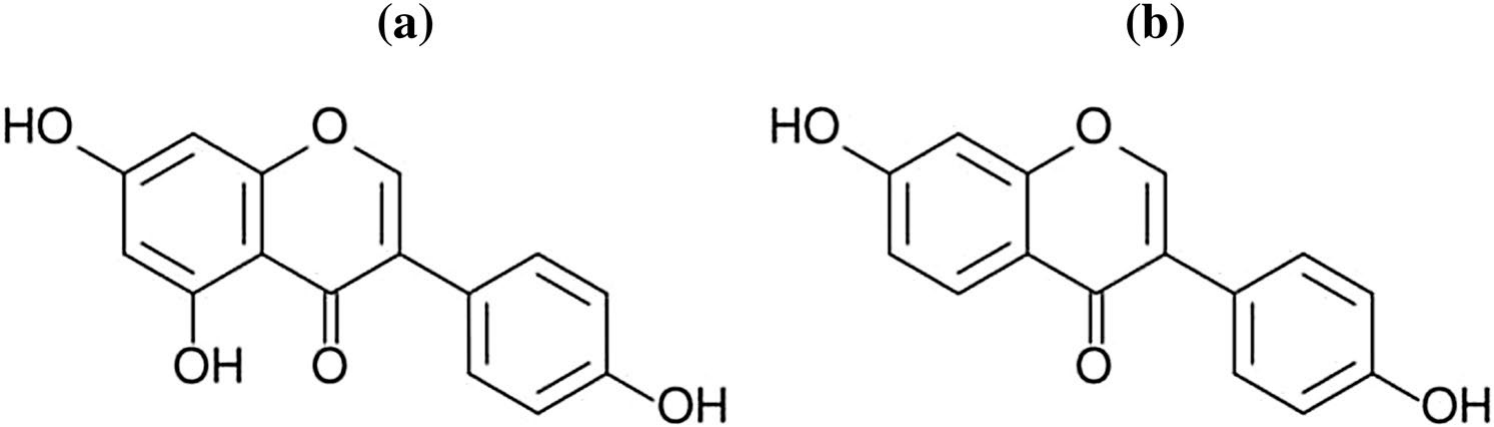
Chemical structure of: genistein (**a**) [230], daidzein (**b**) [220]

**Fig. 9:**
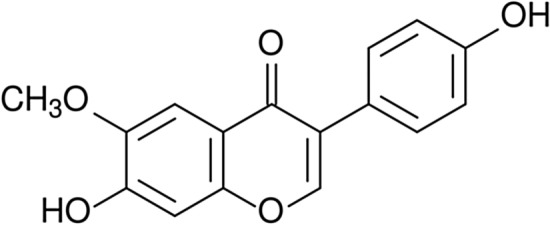
Chemical structure of glycitein [235]

Isoflavonoids occur in various plants: red clover, lentil, spinach, some species of burclover, meadow-grass, coffee beans, plants as the broad bean, and white kwao krua which belong to the bean family [206]. However, soybeans have the highest concentration of isoflavonoids. In the medical industry, the most commonly used isoflavonoids are genistein, daidzein (Fig. 8), and glycitein (Fig. 9) due to their biological activity [207].

Genistein naturally occurs in beans, potatoes, coffee beans, babchi plant, and red clover [208]. This substance inhibits the development of several types of bacteria: *S. aureus*, *H. pylori*, *Bacillus anthracis*, and *Vibrio vulnificus*. Further research does not confirm a similar effect of genistein on *E. coli*, *Lactobacillus reuteri*, *Shigella sonnei*, and *Klebsiella pneumoniae*. It suggests that the biocidal properties of genistein depend on the properties of selected bacteria [209]. Genistein is a natural antioxidant and also antiallergic and anti-inflammatory agent [210, 211].

Genistein encapsulated with biopolymer nanoparticles is a promising material for food and pharmaceutical applications [212–214]. Gelatin and starch could be used as genistein carriers. A high possibility of commercialization of these drug carriers is caused by the low manufacture costs [215, 216]. PLA improves the solubility of genistein. Due to the non-toxicity of PLA and therapeutic properties of isoflavonoid, the pharmaceutical application of their blend is justified [217]. It has been proved that the bioactive properties of genistein contained in gelatin do not change within 3 months. This suggests, that gelatin is an excellent matrix for genistein storage [215]. Chitosan-based nanofibers loaded with genistein could be used for biomedical purposes [218]. Chitosan doped with antioxidant genistein can be potentially used as a functional food additive [219].

Daidzein is another substance that belongs to isoflavonoids. This isoflavonoid occurs in various subspecies of the bean family, red clover, and kudzu roots [220, 221]. The substance exhibits antibacterial and fungicidal properties [222]. The conducted research proved, that daidzein is an auxiliary substance of several antibiotics. *S. aureus* is resistant to methicillin, but its combination with daidzein makes it active against this type of bacteria [223]. The activity of daidzein against *P. aeruginosa* has been proved*.* Moreover, it exhibits antioxidant, anti-inflammatory, and anti-aging properties [220, 224].

Daidzein is a natural adhesive agent which could be applied in certain non-metallic coatings which are widely used in the automotive, furniture, and cosmetic industries [222]. The possibility to improve the adhesion of this type of coatings is crucial due to their low durability and exposure to abrasion during their exploitation. The main reason of the paint coatings chipping is the disruption of their integrity caused by low adhesion between the coating and coated material. The search for an agent which increases adhesion is in the constant process due to the needs of coated products users. The non-toxicity and the renewability are extremely desired in modern materials engineering. Therefore these features of daidzein make it even more attractive as a modern adhesive agent.

Chitosan, starch, gelatin, poly(lactic-co-glycolic) acid (PLGA), and PHB could be used as daidzein carriers which improve its bioavailability [225–229]. This fact shows the pharmacological value of these material blends and daidzein in particular.

Glycitein occurs in soybean and it is responsible for the characteristic taste of products made of soybean [206, 231]. The substance exhibits antibacterial and fungicidal properties against *Colletotrichum gloeosporioides* [232, 233]. It is an antioxidant compound [234]. According to the above-mentioned properties of this isoflavonoid, it has potential applications in various industries (cosmetic, medical, pharmaceutical, and polymer). However, the current literature analysis suggests that glycitein is not well-exanimated yet. Therefore its potential applications as a biopolymer-modifier are poorly studied.

## Blackcurrant Extract

One of the richest sources of polyphenols is a blackcurrant bush. Although the extract obtained from each part of the plant exhibits biocidal properties. The comparison of the amount of the polyphenols in various fruits shows their greater content in the blackcurrant. The content of polyphenols in this plant is 340 mg in 100 g of blackcurrant seeds.

The high content of polyphenols in blackcurrant extract made it a promising modifier of polymer materials due to its non-toxicity and easy cultivation in the middle European climate. Blackcurrant extract is obtained from various parts of the plant such as fruits, leaves, seeds, and buds. However, it has been proved that the blackcurrant buds are the richest source of biocidal substances contained in blackcurrant [236].

There is a wide spectrum of biocidal properties of this extract. It exhibits biocidal activity against *Candida albicans* which belongs to fungi. The mechanism of fungicidal activity is based on fungi cell wall deformation which leads to the leak of the internal substance. This process initiates the inevitable death of the microorganisms [237]. Blackcurrant buds exhibit antimicrobic properties owed to kaempferol, quercetin, rutin, and myricetin contained in them [238]. According to the literature review extract from the blackurrant buds exhibits biological activity against several bacterial strains: *B. subtilis*, *Listeria monocytogenes*, *S. aureus*, *E. coli*, *P. aeruginosa*, and *Acinetobacter bacteria*. The biostatic activity of blackcurrant extract on *C. albicans*, *Alternaria alternata*, and *Aspergillus niger* depends on blackcurrant variety [236, 239]. The effect of the extract on certain fungi strains has been compared to the effect of fluconazole (antibiotic). According to the conducted research, the plant extract is more effective against microorganisms than certain antibiotics [239]. The extract exhibits anti-inflammatory and antioxidant properties due to the content of ascorbic acid which is a natural antioxidant [236, 240]. The blackcurrant berries contain calcium, aluminium, magnesium, and iron which give them their characteristic navy blue colour. The red shade of blackcurrant pulp is caused by the presence of potassium [240]. The pigment substances contained in the blackcurrant berries extract limit its application as modifying additive of biodegradable polymer materials. However, this property can also be an advantage of this modifier in cases where the colour of products is desired. Besides biocidal and antioxidant properties, the extract performs pigment functions which could reduce the number of additives contained in polymer biocomposites. It is another advantage of this extract.

The influence of the blackcurrant extract encapsulated with gelatin on the blood flow has been confirmed. Therefore it can be used for pharmaceutical purposes [241]. The effect of blackcurrant extract on the biopolymer mixtures was investigated. It was founded, that certain blackcurrant concentrations improve the crosslinking, hydrophilic, and optical properties of materials. Hence, this fact makes these materials an interesting solution that could be implemented in the optoelectronic industry [242]. The colouring properties of the blackcurrant anthocyanins are known. The biopolymer-blackcurrant anthocyanins mix is an excellent substitute for the currently used dyes with the synthetic origin [243]. Due to the unique properties (bioactive and antioxidant) of blackcurrant, its complexes with proteins could be applied as an additive in functional food [244]. The starch-blackcurrant complexes have the same application. It has been founded that blackcurrant modifies the physicochemical properties and colour features of the biopolymer [245]. The presence of blackcurrant in gelatin increases its hardness and brittleness which are desired in the food industry [246].

## Tannic Acid

**Fig. 10:**
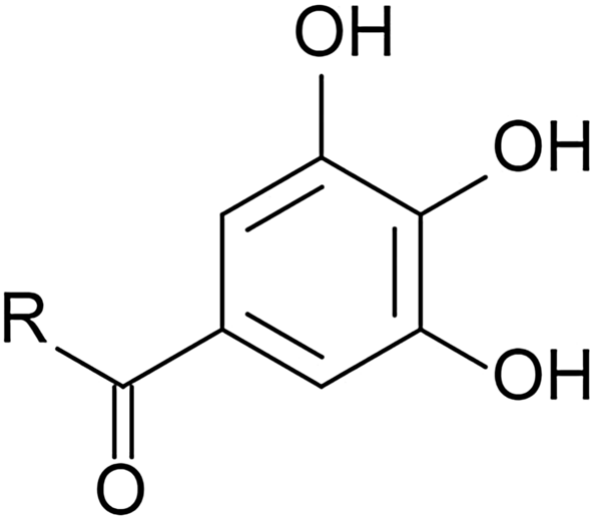
Chemical structure of tannic acid [252, 253]

Tannic acid (Fig. 10) is a substance that occurs commonly in the natural environment as a plant compound [247] This substance is classified as phenol—the organic compound which includes one or more hydroxyl groups connected with the aromatic ring [8]. It is noticed that this substance occurs in almost all aerial parts of plants [247]. The richest source of tannic acid is galls—growths with hardened structures. The galls are appearing as a result of certain insect spices preying and also as by-products produced by mites, fungi, and bacteria. Oak galls, also known as oak apples, are created by two insects that belong to the *Cynipidae* family. One of these insects called the gall-fly is probably the main reason of the galls creation [248]. Besides oaks, galls occur in roses, apples, willows, poplars, beeches, acacias, redwoods, and pistachio trees [249–251]. Tannic acid also occurs in other plants—in the walnut tree bark which is common in middle Europe, in pine and mahogany which grows in Central America. It is the ingredient of strawberries and nettle [251].

Tannic acid exhibits antioxidant properties and is active against viruses, bacteria, and fungi [254–260]. Flu virus and HIV are susceptible to tannic acid. This substance exhibits a bacteriostatic effect against several Gram-negative bacterial strains (*Cytophaga columnaris*, *H. pylori*, *E. coli*, and *K. pneumoniae*) and Gram-positive bacterial strains (*S. aureus* and *L. monocytogenes*, *B. subtilis*) [254–256]. The antimicrobial activity depends on the type of bacteria—the inhibiting effect was stronger against Gram-positive bacterial strains. The tannic acid is also antimicrobic against *C. albicans* which belongs to fungi [254]. Kim [255] proved that the thermal treatment of tannic acid increases its antimicrobic properties.

The combination of several properties (biocidal, antioxidant, and crosslinking) in one additive makes tannic acid a potential modifier that could be applied in polymer biocomposites. When the tannic acid contacts with a solvent, it releases dyes which makes it a natural colouring agent. According to this, tannic acid could replace synthetic dyes which are currently used. The main disadvantage of polymer biocomposites is a low adhesion between phases which could be improved with tannic acid introduction. It increases the mechanical strength of the biocomposite and expands the area of the final product application. The application of tannic acid in biocomposites could probably increase the service life of material due to the reduced amount of microorganisms present on their surface. Moreover, tannic acid could inhibit internal and external antioxidant processes.

This type of biocomposites has a potential application in the medical, catering, and other industries where microbiological hygiene is required. Tannic acid is a promising modifier with biocidal properties which could be applied in the packaging industry due to its non-toxicity. The antimicrobic and antioxidant properties of tannic acid are confirmed with research conducted on the biodegradable packaging made of starch doped with this substance [256]. This type of packaging can certainly be implemented in the industry and have the potential to replace the currently used packaging made of crude oil. Polymer biocomposites made of chitosan containing tannic acid are interesting materials with potential application in cosmetology as acne patches [257]. The colonization of the sebaceous glands by the bacteria *Propionibacterium acnes* is one of the reasons of acne [261]. The biocomposites with tannic acid probably could lead to the death of these pathogenic microorganisms. However, this type of research is not done so far and the effect of tannic acid on these bacterial strains is not investigated yet.

These complexes could be applied in drug or supplement delivery systems as a carrier due to the non-toxicity and ability to controlled supplement/drug release [262]. Gelatin microspheres containing tannic acid could be applied for nutraceutical purposes [263]. Gelatin/tannic acid films exhibit antibacterial properties which are desired in biomedical materials [264]. Gelatin/tannic acid hydrogels with shape memory are interesting materials for potential biomedical and robotic applications [265]. Biocomposite nanofibers with tannic acid could be used as a biocompatible wound dressing material with an antibacterial effect [266]. The zein/tannic acid complexes acid have a wide range of applications in food and cosmetic industries due to their stabilizing and crosslinking properties [267, 268]. Zein/tannic acid coatings elongate the shelf life of fruits, therefore this kind of edible layers could be used as non-toxic biopreservatives in the food industry [269]. Zein particles modified with tannic acid improve mechanical properties and hydrophobicity of gelatin-based composites for food packing [270]. The gelatin doped with silver nanoparticles and tannic acid is another material for potential food packing application [271]. The chitosan films containing tannic acid could have the same application [272]. Tannic acid improves interfacial adhesion in composites and as a result—mechanical properties of ones. Therefore, it could be used in polymer biocomposites production [273, 274]. Chitosan/tannic acid coatings applied on biocomposites fillers increase the fire resistance of biocomposites. This kind of coatings has a potential application in materials engineering [275].

## Betulinic Acid

**Fig. 11:**
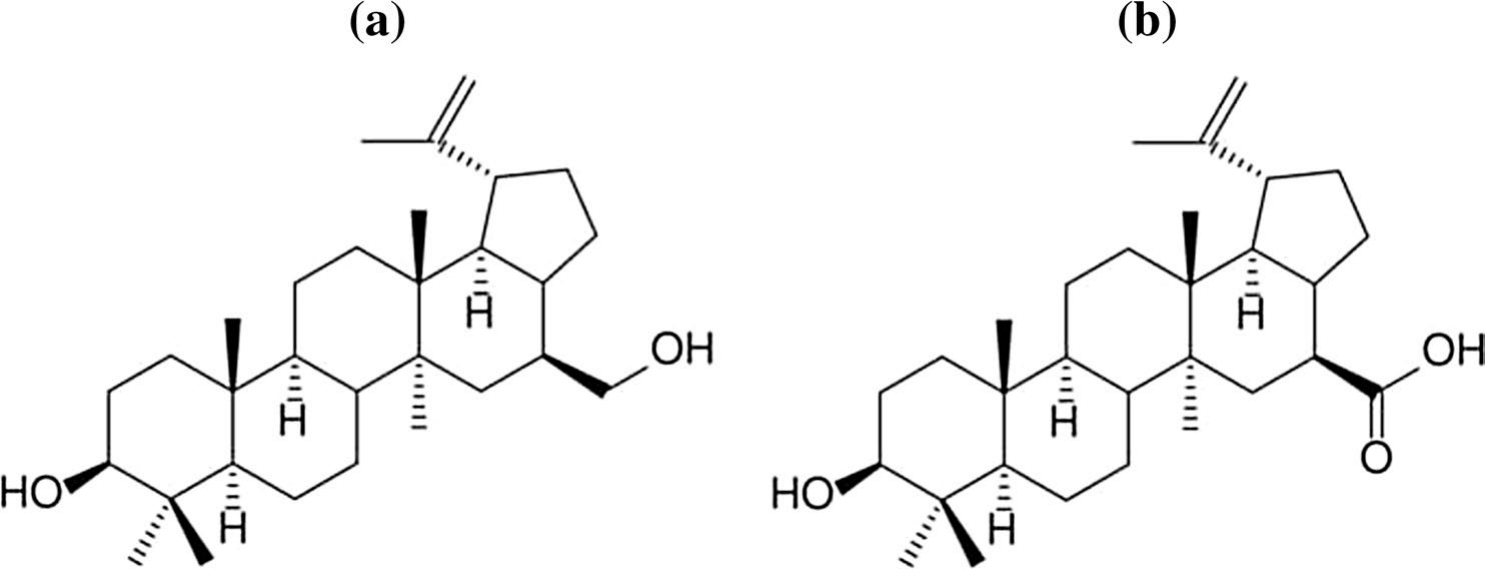
Chemical structure of: betulinic acid (**a**) and betulin (**b**) [282, 283]

Betulinic acid belongs to triterpenoids which are the triterpenes derivates. The source of the betulinic acid is i.a. varied birch species [276, 277]. This substance is obtained from betulin (BE) shown in Fig. 11. The betulin occurs in plants such a birch, London plane tree, jujube, Caucasian alder, thistle, and rosemary [278, 279]. Among mentioned plants, the highest concentration of betulin is found in birch bark which consists of inner and outer parts. It has been proved, that the content of betulin in the outer bark ranges from 30 to 35% and this value depends on the birch variety [276, 280]. The white colour of the tree is caused by the presence of betulin, which is a natural dye. This substance was discovered in 1788 by a pharmacist and chemist Tobias Lowitz [281].

Betulinic acid exhibits antiviral properties against HIV due to the functional groups that occur in it at carbon atoms C-23 and C-28. Betulinic acid is biocidal against the *Herpes simplex* virus (type 1 and type 4) and several *Enterovirus* viruses [284–286]. Some of the betulin derivatives are also exhibiting antiviral activity. Bevirimat which is produced by the chemical modification of betulinic acid exhibits the highest activity against HIV and inhibits HIV-1 and HIV-2 [287]. This substance also inhibits the advanced stage of HIV-1. Due to the mentioned properties of bevirimat, it is a promising compound that could be used as an anti-HIV drug [279, 288].

Antibacterial property is another advantage of betulinic acid. According to conducted research, the tannic acid is the only betulin derivate with biostatic activity against *E. coli*, *P. aeruginosa*, and *Enterobacter aerogenes*. It is probably caused by the structure of mentioned bacteria which belong to Gram-negative bacteria. They have an extracellular membrane which could be an obstacle during the penetration of active compounds—betulin derivatives. Furthermore, betulinic acid exhibits biostatic activity against *E. faecalis* bacterial strain and inhibits the growth of these bacterial colonies by 56% [289]. The antibacterial activity of betulinic acid against bacteria *P. aeruginosa*, *E. coli*, and *S. aureus* bacterial strains is caused by increased production of superoxide anion radicals which cause oxidative stress in bacterial cells. This process is unfavorable for bacterial cells and most often leads to their death. The oxidation process of bacterial cells has been proved by increased concentration of malondialdehyde which is an indicator of oxidative stress and cell destruction [290]. According to the current literature state, the substance also inhibits the SARS-CoV virus [291]. The inhibiting mechanisms of viruses can be divided into two groups which are implemented at different stages of viral development. The processes which belong to the first group are activated during the cell penetration by the virus. These processes make it difficult for the virus to cross the cell membrane. The second group is based on inhibiting the process of viral replication caused by the effect of betulinic acid on SARS-CoV 3CL protease [291]. Ascorbic acid is a substance that has a synergistic effect on betulinic acid.

The literature overview allowed us to estimate the validity of betulinic acid application as a modifier of biodegradable polymer materials. The estimation was based on the previously described properties of the substances. Betulinic acid is a new substance in the polymer industry, therefore the information about its behavior and effects on the polymer matrix is very limited. However, there is a reliable report—a patent invented by scientists from the University of Silesia in Katowice, Poland, and the Medical University of Silesia in Katowice. The method of obtaining betulin-modified thermoplastic polymers is the subject of this patent. The characteristic features of this polymer are antibacterial and anti-inflammatory properties [292]. This is a breakthrough discovery because betulin and betulinic acid have similar properties. This fact suggests that polymer materials containing betulinic acid would probably exhibit antibacterial properties as well. This will undoubtedly extend the service life of final products and increase the area of their potential application. Such areas may be industries where sterile conditions are desired.

The effectiveness of betulinic acid against parasites has been proved via testing chitosan nanoparticles loaded with betulinic acid [293, 294]. Certain biopolymer coatings containing betulinic acid exhibit anticancer properties [295–297]. The same properties were noticed in biopolymer-based nanoparticles loaded with betulinic acid [298, 299]. Therefore, the pharmaceutical potential of betulinic acid is huge. From the literature overview, it could be concluded, that the applications of betulinic acid as a biopolymer-additive in other industries have not been described yet.

## Lapachol

**Fig. 12:**
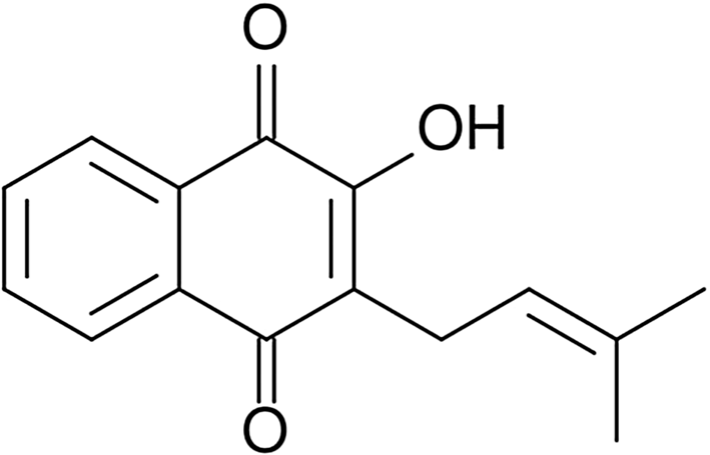
Chemical structure of lapachol

Lapachol is a compound that occurs both in the inner part of the bark and in the heartwood of *Tabebuia* trees, commonly found in South and Central America [300]. In the colloquial language of Brazilians, this tree species is also called taheebo, pau d’Arco, or lapacho which probably gives the name to the active substance contained in the tree bark [301, 302]. According to the literature reports, the other plants which belong to the *Bignoniaceae* family (as well as *Tabebuia* does) also include lapachol in their timber [303, 304]. The *Tabebuia* bark has been already used by Incas in ancient times—the infusion of chopped tree bark was applied for medicinal purposes. In 1882 lapachol was extracted by Italian phytochemist—Emanuel Paterno for the first time [301, 302, 306, 305]. Lapachol (2-Hydroxy-3-(3-methyl-2-butenyl)-1,4-naphthoquinone) is classified as naphthoquinone [303]. Naphthoquinones are organic compounds derived from naphthalene. The presence of ketone groups (C=O) in the naphthoquinones structure is known [8, 307]. The chemical structure of lapachol is illustrated in Fig. 12.

Lapachol exhibits biological activity against microorganisms. Its biocidal mechanism is based on the initiation of oxidation processes and enzyme inhibition. Both reactions occur in cells [300]. It has been proved that the biological activity of naphthoquinones depends on their structure [308]. The biological effect of this substance is similar to the effect of antibiotic amphotericin B. Lapachol is effective against the following bacterial strains: *H. pylori*, *Streptococcus*, *Enterococcus*, *Clostridium*, *Staphylococcus*, and *Bacillus* which are hazardous for human health. The antifungal activity extends to *Candida* species and *Cryptococcus neoformans* [300]*.* Lapachol like the majority of naphthoquinones exhibits colouring properties and could be used as the yellow pigment [301, 303, 304]. It is also a natural antioxidant agent [309].

The unique properties of lapachol and its toxicity against microorganisms are advantages. The resistance of polymer materials on the microorganisms is crucial due to their common exposure to the microorganisms. Hence, the bark of *Tabebuia* which contents lapachol is a promising material that could be introduced in biodegradable polymer biocomposites. The modification of biocidal and mechanical properties of biocomposites with the lapachol does not pollute the environment. The biocomposite that consists of the biodegradable PLA matrix and the reinforcement such a *Tabebuia* bark is completely biodegradable. This type of biocomposite has been examined by authors. The manufacturing of the biocomposite was carried out in several stages—by extrusion with granulation and injection. The enzymatic biodegradation studies were performed according to the method contained in the article [310]. These studies lasted 8 weeks. Due to our studies [311], the increase of bark content in biocomposite leads to the increase of the percent mass loss which proves higher biodegradability of biocomposite. The same dependence has been noticed during the mechanical studies. The increased content of *Tabebuia* bark in the biocomposites increases the tensile modulus of materials. The presence of lapachol contained in the *Tabebuia* bark improved the thermal durability of the biocomposites. However, more detailed research of lapachol influence on the thermal properties of PLA is recommended. According to biocidal studies, the antimicrobic activity of biocomposites was lower than the one mentioned in [301]. The decrease of biocidal activity of the biocomposites is caused by the high processing temperatures (e.g. extrusion and injection molding) applied.

The lapachol derivative—lapachol sodium salt exhibits biological activity and could be used as a drug. The chitosan flakes/lapachol sodium salt complex increases the bioavailability of the latter [312]. The lapachol derivative lapazine has a potential application as a drug used in infectious diseases treatment. The studies of alginate/chitosan microparticles loaded with lapazine prove it. β-Lapachone is another lapachol derivative with a wide range of therapeutic properties. However, the high toxicity of β-lapachone is an obstacle to its implementation into the pharmaceutical industry. The studies on the β-lapachone connected with chitosan confirms the decrease of agent toxicity. This fact increases the probability of its application in the pharmaceutical industry [313, 314]. According to Pereira et al. [315], starch could be used as a lapachol carrier in drug delivery systems.

## Allicin

**Fig. 13:**
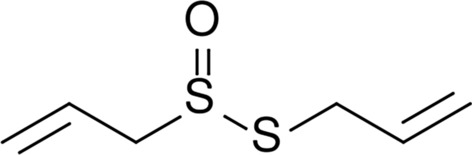
Chemical structure of allicin [319]

Allicin (diallyl thiosulfonate) (Fig. 13) is the main ingredient of garlic, onion, and clove extracts which exhibits biocidal properties [8, 316]. It creates during garlic or onion crushing [317]. This compound belongs to phytoncides—the bioactive compounds that are produced by selected plants. Phytoncides are defined as natural antibiotics. The biocidal properties of garlic extract were observed at the end of the nineteenth century by Louis Pasteur. The isolation of allicin from cloves was carried out by Chester John Cavallito and John Hays Bailey in 1944 for the first time [318]. The highest concentration of allicin is found in the garlic extract. For this reason, the plant is applied in traditional medicine. Moreover, due to its high taste attributes it is used in almost every cuisine of the world. The wide spectrum of garlic biocidal properties has caused a growing interest in the modern scientific world including materials engineering.

Allicin is a substance that exhibits a strong antioxidant effect based on free radicals inhibiting [318, 320]. It has antifungal and antimicrobic (the antibacterial activity against several Gram-positive and Gram-negative bacterial strains was noticed) properties [319, 321, 322]. The mechanisms of the biocidal action of the substance are not well known so far, but literature reports show that the formation of allylsulfide compounds changes l-cysteine, which is a free amino acid [318]. Allicin has a cytotoxic effect on proteins contained in microbes cells. The penetration of parasitic cells by allicin leads to their death [323].

According to the conducted research, the highest activity of allicin (more than 86%) was noticed on the 3rd day of studies while this percentage value changed over time. The last measurement has been done on the 11th day of studies and the percentage value of substance activity decreased by almost 1/3 compared to the 3rd day of studies. It was probably caused by the high volatility of allicin. However, the substance had high biological activity even after 11 days of exposure to the bacteria [324]. The allicin is also bioactive against several fungi and protozoa [325].

Due to the wide spectrum of biocidal properties of allicin, it can replace currently used additives which give the resistance of the biodegradable material to the adverse effects of pathogenic microorganisms. However, one of the main disadvantages of this compound is the characteristic sulfuric smell which is associated with the garlic smell. According to this, allicin can be applied in biodegradable materials which have limited contact with humans. It can also be used in conditions where the controlled development of microorganisms on the surface of the material is desired.

Cellulose nanoparticles doped with allicin exhibit antimicrobial properties which suggest that this complex could be used in food, food packing, and textile industries to limit microbial proliferation [326]. The starch-based wall material is used to produce allicin microcapsules which can be used as a biopreservative in the food industry [327]. The chitosan/allicin complex exhibits grown antimicrobial activity, therefore this material is suitable for the food industry applications [328–330]. Allicin encapsulated in chitosan/starch could be used as a nitrogen fertilizers additive. The presence of allicin elongates the release of the nutrients in the soil which is desired in perennial plants cultivation [331]. Gelatin nanoparticles loaded with allicin could be used in cancer therapy due to the anticancer activity of allicin [332]. The strong antibacterial activity of biocomposites [chitosan/polyvinyl alcohol (PVA)] doped with allicin has been noticed. The long-term antibacterial impact makes allicin a perspective material for medical purposes as a tissue engineering and wound dressing material [333, 334].

## The Effect of Modifiers on Certain Biopolymers

The modification of biopolymers is one of the basic steps in their processing. It helps to suit the biopolymers to certain applications. The aim of the modification is based on changing, improving, or/and creating new properties of the materials. The below table summarizes the modification effects of biopolymers caused by plant-based modifiers (Table 1).

**Table 1:** The effectiveness of the biopolymers modification by certain natural additives

Modifier	Material	Modification effects
Lignin	PLA	Enhanced thermal resistance [335]
Caffeic acid, gallic acid	Gelatin	Increased mechanical and antioxidant properties [336]
Vanillic acid	PLA	Improved resistance on the photooxidative degradation [113, 337]
Silymarin	PLA/PHB blends	Enhanced resistance on thermo-oxidative degradation [119]
Resveratrol	PLA	Improved photo-oxidative and thermal stability [127]
Hesperidin	PLA, PHA	Improved oxidation resistance [148]
Apigenin	Starch	Decreased digestion rate of and improved thermal stability [165]
Kaempferol, myricetin, quercetin	Chitosan	Improved mechanical properties, reduced oxygen and water vapor permeability, decreased UV light transmittance [205]
Quercetin	Gelatin	Increased mechanical properties and decreased swelling degree, improved the UV-light absorption [336, 338]
	Chitosan	Reduced transparency and altered tint (to green one) [189]
	Starch	Elevated thermal stability [196]
	PLA/PEG blends	Enhanced mechanical and thermal properties, changed colour and reduced transparency [194]
	PLA	Improved resistance on the photooxidative degradation [337]
Blackcurrant	Starch	Altered colour and physicochemical characteristics [245]
	Gelatin	Increased hardness and brittleness of polymer [246]
Tannic acid	Zein	Changed shape of zein molecule which affects wettability changes [267]
	Gelatin	Improved mechanical properties, increased compatibility between polymer matrix and additives modified with tannic acid, improved antioxidant activity, stability, transparency, and antibacterial properties [263, 270, 339]
	Gelatin/silver nanoparticles	Synergistically increased antibacterial properties [271]
	Chitosan	Improved transparency, antibacterial properties; increased tensile strength and decreased solubility of the material, affected synergistically on plasticizer contained in the material [272, 340]
	PLA/filler	Improved adhesion between polymer matrix and filler and greater dispersion of filler in the matrix [273, 274]
Betulinic acid	PEG	Changed physical structure [341]
Lapachol	PLA	Increased thermal durability and biodegradability [311]
Allicin	Chitosan	Increased water solubility and changed colour [328]
	Chitosan/PVA blend	Decreased hydrophilicity, increased porosity and changed microstructure [333]

## Conclusions

This literature overview shows a new direction in the development of natural modifying substances with biocidal properties. The compounds contained in plants are an undoubtedly competitive group of natural modifiers because the effects of their antimicrobial activities are comparable to those of some synthetic biocides. In some cases, the natural compounds exhibit stronger biocidal activity. This fact makes them an interesting alternative for synthetic modifiers. Non-toxicity and complete biodegradability are some of their unquestionable advantages. Further development of natural modifiers and focus on biocidal properties of polymer materials are expected. Those expectations are justified due to the current pandemic conditions and the necessity of the elongated service life of the biocomposites. The biodegradability of polymer materials and their modifiers is crucial. Hence, the environmentally friendly and non-toxic modifiers are in constant search.

## Data Availability

Data is contained within the article.
